# Qualitative and Quantitative Assessment of Vascular Changes in Diabetic Macular Edema after Dexamethasone Implant Using Optical Coherence Tomography Angiography

**DOI:** 10.3390/ijms18061181

**Published:** 2017-06-02

**Authors:** Lisa Toto, Rossella D’Aloisio, Marta Di Nicola, Giuseppe Di Martino, Silvio Di Staso, Marco Ciancaglini, Daniele Tognetto, Leonardo Mastropasqua

**Affiliations:** 1 Ophthalmology Clinic, Department of Medicine and Science of Ageing, University “G. D’Annunzio” Chieti-Pescara, Chieti 66100, Italy; l.toto@unich.it (L.T.); mastropa@unich.it (L.M.); 2 Department of Medicine, Surgery and Health Sciences, University of Trieste, Trieste 34100, Italy; tognetto@units.it; 3 Department of Medical, Oral and Biotechnological Sciences, Laboratory of Biostatistics, University “G. D’Annunzio” Chieti-Pescara, Chieti 66100, Italy; mdinicola@unich.it; 4 School of Hygiene and Preventive Medicine, Department of Medicine and Science of Ageing, University “G. D’Annunzio” Chieti-Pescara, Chieti 66100, Italy; peppinodimartino@hotmail.com; 5 Ophthalmology Clinic, Department of Life, Health and Environmental Sciences, University of L’Aquila, L’Aquila 67100, Italy; silvio.distaso@cc.univaq.it (S.D.S.); marco.ciancaglini@cc.univaq.it (M.C.)

**Keywords:** optical coherence tomography angiography, diabetic macular edema, intravitreal dexamethasone implant, diabetic retinopathy

## Abstract

The aim of this study was to investigate retinal and choriocapillaris vessel changes in diabetic macular edema (DME) after the intravitreal dexamethasone implant (IDI) using optical coherence tomography angiography (OCTA). Moreover, a comparison between morphological and functional parameters of DME and healthy patients was performed. Twenty-five eyes of 25 type 2 diabetic retinopathy patients complicated by macular edema (DME group) and 25 healthy subjects (control group) were enrolled. Superficial capillary plexus density (SCPD) and deep capillary plexus density (DCPD) in the foveal and parafoveal areas, choricapillary density (CCD) and optic disc vessel density (ODVD) were detected using OCTA at baseline and after 7, 30, 60, 90 and 120 days post injection. Best corrected visual acuity (BCVA), retinal sensitivity, and central retinal thickness (CMT) were also evaluated in both groups of patients. A statistically significant difference between the two groups (DME and controls) was found in terms of functional (MP, *p* < 0.001 and BCVA, *p* < 0.001) and morphological (CMT, *p* < 0.001; SCPD in the parafoveal area, *p* < 0.001; DCPD in the foveal area, *p* < 0.05 and parafoveal area, *p* < 0.001; CCD, *p* < 0.001) parameters. After the treatment, SCPD and DCPD in the foveal and parafoveal areas did not modify significantly during the follow up.

## 1. Introduction

Diabetic retinopathy (DR) is one of the leading causes of vision loss and blindness in the working age population [1].

Regardless of the widespread use of new therapies, including the anti-vascular endothelial growth factor (VEGF) agents and corticosteroids, diabetic macular edema (DME) remains a common DR complication, and it is estimated to affect about 20% of the diabetic retinopathy patients in the United States [2].

The DME etiology is still being investigated, and it is probably related to an inflammatory condition [3].

Capillary leakage and fluid accumulation, due to a breakdown of the blood-retinal barrier, involve the expression of inflammatory factors, such as intercellular adhesion molecule-1, interleukin-6, monocyte chemotactic protein-1, leukostasis and VEGF [3].

In the armamentarium of intravitreal agents, the anti VEGFs were the first drugs approved for diabetic macular edema treatment [4].

Nevertheless, the intravitreal dexamethasone implant (IDI) is considered an effective corticosteroid in the treatment of DME. It has been demonstrated that IDI is six times more effective than intravitreal triamcinolone acetonide [5,6].

Together with the development of modern drugs for DME treatment, new imaging techniques are now part of the routine follow up of the diabetic retinopathy (DR) patients.

Nowadays, the introduction of optical coherence tomography angiography (OCTA) allows for the precise and detailed visualization of retinal and choroidal blood vessels without using injectable dyes, and it has been used for the diagnosis and follow up of several vascular retinal diseases [7,8].

Although Fluorescein angiography (FA) is still the gold standard for the diagnosis and the evaluation of retinal microvascular alterations, it has some limitations in the optimal visualization of deep retinal vessels [9].

The aim of this prospective study was to evaluate morphological macular and peripapillary retinal vascular changes in patients with DR complicated by DME before and after IDI using XR Avanti^®^ AngioVue OCTA (Optovue Inc., Fremont, CA, USA) based on the split spectrum amplitude decorrelation angiography (SSADA) algorithm, and to compare them with functional parameters. In addition, a comparison between morphological and functional parameters of DME patients and healthy subjects was performed.

## 2. Results

### 2.1. Demographic Data

A total of 50 patients were enrolled in this study from June 2016 throughout January 2017.

Twenty-five eyes of 25 type 2 diabetic patients (DME group) (13 males; 12 females; mean age of 62.3 ± 8.3 years) with DME treated with IDI were evaluated for the analysis.

All diabetic subjects were treatment naïve patients.

A control group of 25 healthy patients (control group) (11 males; 14 females; mean age of 61.8 ± 6.8 years) was also enrolled.

No treatment-related complications were observed during the follow up, including intraocular pressure increase requiring hypotonic eye drops and progression of lens opacity evaluated by means of Lens Opacities Classification Systems (LOCS III) [10,11].

### 2.2. Functional Parameters at Baseline

The mean Best corrected visual acuity (BCVA) and 4° microperimetry (MP) values are reported in Table 1.

A statistically significant difference was found between the two groups of patients in terms of functional parameters (MP and BCVA).

The mean BCVA at the baseline was 0.6 ± 0.1 logMAR in the DME group and 0.1 ± 0.1 logMAR in the control group (*p* < 0.001) (Table 1).

The mean microperimetry sensitivity at the baseline was 4.9 ± 4.3 dB in the DME group and 15.0 ± 1.4 dB in the control group (*p* < 0.001) (Table 1).

### 2.3. Morphological Parameters at Baseline

A statistically significant difference was found between the DME group and control group in terms of morphological parameters (Table 1).

The mean central macular thickness (CMT) in the foveal area at the first visit was 462.1 ± 109.6 μm in DME group and 252.7 ± 20.0 μm in the control group (*p* < 0.001) (Table 1).

The mean superficial capillary plexus (SCPD), the mean deep capillary plexus density (DCPD), the mean choriocapillaris density (CCD), and the mean optic disk vessel density (ODVD) are described in Table 1.

Overall, retinal vessel density was significantly reduced in DME patients in comparison with controls (Table 1).

Superficial retinal vessels in the macular area were diffusely rarified with disruption of perifoveal anastomotic arcades. In the deep retinal plexus, a rarefaction of vessel density was observed with telengectatic appearance of retinal vessels particularly in the areas of macular edema.

Vessels of the optic disc were not statistically different between the two groups (*p* = 0.431) (Table 1), while peripapillary vessel density was significantly reduced in the DME group compared to the control group (*p* = 0.021).

Chorocapillaris density at baseline was significantly reduced in DME group in comparison with control group, both in the foveal and parafoveal areas (Table 1).

### 2.4. Post Treatment Qualitative and Quantitative Analysis

CMT in the foveal area significantly decreased at the postoperative controls from 462.1 ± 109.6 μm to 305.8 ± 53.9 μm at 30 days and 286.7 ± 53.9 μm at 60 days. After 90 days from the implant CMT started to increase to 307.8 ± 60.1 μm and reached 426.0 ± 95.7 μm 120 days after the treatment (Table 2).

Overall, SCPD, DCPD and CCD did not modify significantly during the follow up.

Nevertheless, CCD showed a weak increasing trend from 60.7 ± 5.8 to 64.7 ± 5.8 at 60 days in the foveal region.

**Figure 1:**
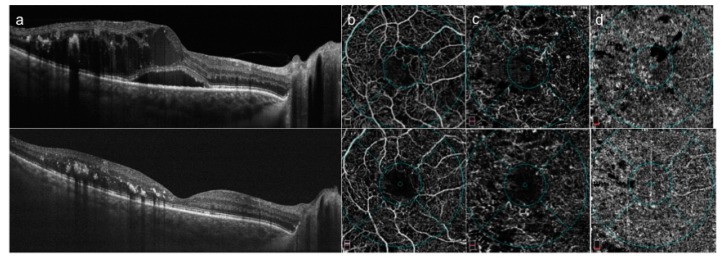
Spectral optical coherence tomography (SDOCT) and OCT angiography (OCTA) images of a patient with diabetic macular edema before and 60 days after intravitreal dexamethasone implant (IDI). SDOCT horizontal scan at baseline ((**a**): top) showing intraretinal cystoid macular edema and subretinal fluid that almost completely resolved after IDI implant ((**a**): bottom). OCTA of superficial plexus ((**b**): top) showing reduction of superficial vessel density and interruption of perifoveal anastomotic arcades with partial increase of vessel density after edema disappearance at 60 days after IDI implant ((**b**): bottom). OCTA of the deep plexus at baseline ((**c**): top) showing telangiectatic and rarified vessels and no flow areas related to retinal cysts and hard exudates; after IDI implant ((**c**): top) it is evident a reduction of vessel caliber appearing less angiectatic, a vessel density increase and partial cysts disappearance. OCTA of choriocapillaris ((**d**): top) at baseline showing focal areas of no flow probably related to a masking effect due to retinal edema and hard exudate that partially decrease after IDI ((**d**): bottom).

At qualitative analysis, after partial or complete resolution of macular edema, superficial and deep retinal vessels were still rarefied, but in the deep plexus vessels appeared less telangectatic (Figure 1).

During the entire follow up period, the ODVD did not show any statically significant difference (Table 2).

The retinal sensitivity at microperimetry significantly improved at 7, 30, and 60 days after the IDI with stable values up to 90 days and a slight not significant decrease at 120 days.

BCVA showed a tendency to an increase with the highest value at 60 days remaining stable thereafter (0.3 ± 0.2 logMAR). 

### 2.5. Correlation Analysis between Different Parameters

SCPD and DCPD relative variation from baseline to 120 days in the foveal and parafoveal area did not show any significant correlation with foveal and parafoveal CMT relative variation (Table 3).

A significant negative correlation was found between CCD relative variation and CMT relative variation in foveal and parafoveal areas (respectively, *r* = −0.724; *p* < 0.01 and *r* = −0.799; *p* < 0.01).

## 3. Discussion

In this study, using OCTA, we investigated retinal superficial and deep vessel densities and choriocapillaris density in patients with diabetic retinopathy complicated by macular edema at baseline and after an intravitreal dexamethasone implant. Overall, we found a reduction of foveal and parafoveal retinal superficial and deep vascular density, and choriocapillaris density compared to normal controls. In addition, peripapillary vessel density was also decreased compared to controls. Retinal superficial and deep vessel density did not change significantly after intravitreal dexamethasone implant; on the contrary, choroid vessel density showed a tendency toward an increase.

The spread of OCTA allowed for a better assessment of the microvascular retinal alterations in DR and DME patients. Some previous studies have reported retinal capillary network and choriocapillaris abnormalities in patients with diabetic retinopathy, such as a decrease of vessel density, with a significant decrease of capillary perfusion density values as retinopathy progresses [12,13,14,15]. The reduction of vessel density was more consistent in the deep capillary plexus and choriocapillaris compared to the superficial plexus [12,15].

Similarly, in our study, the vessel density of both superficial and deep capillary plexuses of the DME patients was lower than in the healthy controls with DCPD being much more affected than SCPD.

Superficial capillary plexus density was significantly reduced in the parafoveal area (*p* < 0.001), while deep capillary plexus and choriocapillaris densities were significantly reduced both in the foveal (*p* < 0.05) and parafoveal area (*p* < 0.001 and *p* < 0.01, respectively).

At qualitative analysis the superficial capillary plexus was rarified with interruption of the perifoveal anastomotic arcade, and deep plexus was rarified with evidence of telangectatic vessels particularly in areas of macular edema. In addition, peripapillary vessel density is significantly decreased in DME eyes compared to controls (*p* < 0.05).

In reference [12], it has been speculated that integrity of the DCP could be a possible predictor of the effectiveness of the treatment, probably related to its role in excess fluid removal from the retina, thus preserving it from macular edema. In fact, Junyeop et al. found a significant correlation between the status of the DCP and the treatment response. The DCP of poor responders showed greater damage, such as a lower vascular flow density, a higher mean number of microaneurysms, and a larger foveal avascular area in comparison with the good responders.

They did not find any association between flow density of the superficial retinal vessels and retinal thickness before and after the treatment [12].

In the current study, no correlation was found between SCPD/DCPD and CMT at baseline.

After the IDI implant, the SCPD and DCPD in the foveal and parafoveal area did not modify significantly during the entire follow up, although retinal macular thickness significantly decreased.

It has been demonstrated that intravitreal steroid, such as dexamethasone or triamcinolone in eyes with macular edema due to retinal vein occlusion or diabetic retinopathy, causes a reduction of arteriolar or venular vessel diameter probably due to a blockage of vascular endothelial growth factor with macular edema improvement [16,17].

At a subjective evaluation, we observed a normalization of vessel caliber after retinal edema resolution with deep vessels being less angiectatic; nevertheless, at a quantitative vessel density analysis, we did not find a significant change of vessel density both in the superficial and deep plexuses. This is probably due to the ischemic damage of retinal vessels that do not recover after treatment.

The CCD after treatment showed a weak tendency to an increase. We can hypothesize that overlying pathologic retina due to edema could attenuate the OCT signal with choriocapillaris appearing reduced.

As already reported by Spaide, en face imaging that relies on segmentation strategies is problematic in diseased eyes due to image artifacts. In cases of retinal edema, retinal segmentation can be problematic due to an increase of layer thickness and contemporary decrease of contrast between different layers causing automatic recognition of retinal layers and resulting segmentation based on segmentation of healthy retina to fail. In addition, the increase in thickness of retinal layers could reduce the signal strength of the underlying choroid, thus causing a misleading vessel density analysis [18].

In the DME group, peripapillary vessel density was significantly reduced compared to that of healthy subjects. Peripapillary vessels’ features in patients with diabetic retinopathy have been previously investigated in the literature with other imaging techniques. Hamanaka et al. evaluated retinal ischemia in the peripapillary area in patients with proliferative diabetic retinopathy using fluorescein angiography, and correlated the extension of peripapillary capillary occlusion to angle neovascularization [19]. Moradi et al. showed that wider baseline peripapillary retinal venular caliber may be a predictor of better visual outcome in DME eyes treated with anti-VEGF, probably related to intraocular level of VEGF [20].

Recently, Rao et al. investigated peripapillary vessel density in patients with systemic diseases such as diabetes and systemic hypertension using OCTA. They observed a lower peripapillary vessel density in diabetics than in healthy controls [21]. Our results confirm this finding; nevertheless, we evaluated only patients with moderate DR complicated by DME, thus it is not possible to generalize these results to all stages of DR with or without DME, and establish a relationship with DME.

Functional parameters such as visual acuity and microperimetry increased after IDI implant related to macular thickness decrease.

A trend toward an improvement in visual acuity was observed after IDI with the highest gain at 60 days after the treatment. Retinal sensitivity detected with microperimetry significantly improved, as soon as the 7-day control reached the lowest value at 60 days, and remained stable at 90 days with an increase at 120 days after IDI, not reaching the preoperative values.

The effect peak of the dexamethasone implant has been previously reported to be at 30 days with a mean duration of the treatment being at four months [22].

Shah et al. reported that also in vitrectomized eye implantation of the IDI appeared to be efficacious in improving visual acuity and CMT with the observed benefits persisting for at least for three months [23].

## 4. Materials and Methods

### 4.1. Study Participants

Twenty-five type 2 diabetic retinopathy patients with a moderate stage of DR (according to the simplified version of the Early Treatment of Diabetic Retinopathy Study (ETDRS) classification aimed by the American Academy of Ophthalmology Guidelines Committee) complicated by center-involved DME and candidates to IDI were enrolled in this prospective study [24]. The diagnosis of DR was made by means of fundus examination. In addition, fluorescein angiography (FA) and Spectral optical coherence tomography (SD-OCT) were performed in all cases.

Criteria for inclusion were: (1) age >18 years old; (2) best-corrected visual acuity (BCVA) greater than 0.5 logMAR in the study eye at baseline examination; (3) presence of treatment naïve recent center-involved DME (less than 3 months); (4) central macular thickness (CMT) > 300 µm as measured using SD-OCT at the baseline examination.

The exclusion criteria were: (1) any previous ocular surgery (included intravitreal injections) in the last 6 months; (2) laser treatments; (3) history of glaucoma and other conditions to contraindicate steroid treatment; (4) retinal vascular diseases; (5) medium lens opacities (according to the Lens Opacities Classification System).

For each patient, the eye with lowest BCVA was selected, and in the case of equality of BCVA between the two eyes, the patient was given the choice.

Twenty-five age-matched healthy subjects were included as controls. The study adhered to the tenets of the Declaration of Helsinki and was approved by the Ethical Committee “Department of Medicine and Science of Aging, University “G. D’Annunzio” Chieti-Pescara, Italy” (LED, v01, February 2016). Written informed consent was provided for all the patients enrolled in the study.

### 4.2. Study Protocol

All recruited patients underwent a complete ophthalmic evaluation, including assessment of BCVA, tonometry, slit-lamp biomicroscopy, and indirect fundus ophthalmoscopy.

BCVA was assessed using the Early Treatment Diabetic Retinopathy Study (ETDRS) chart.

All patients were tested by means of XR Avanti^®^ AngioVue OCTA (Optovue Inc., Fremont, CA, USA).

FA images previously acquired with Heidelberg Retina Angiograph 2 HRA2 (HRA+OCT Spectralis: Heidelberg Engineering, Heidelberg, Germany) were used to confirm the stage of the diabetic retinopathy by two independent experienced retina specialists (LT and RDA).

All of the patients with no ocular or systemic contraindications to steroid treatment and signing an informed consent were treated with a sustained-release dexamethasone 0.7 mg intravitreal implant (IDI; Ozurdex^®^, Allergan, Inc., Irvine, CA, USA) within 7 days from baseline examination.

### 4.3. Procedures

#### 4.3.1. SD-OCT Angiography with XR Avanti

XR Avanti^®^ AngioVue OCTA (Optovue Inc., Fremont, CA, USA) is a device with a high-speed of 70,000 axial scans per second, using a light source of 840 nm, and an axial resolution of 5 μm. The AngioVue OCTA system, based on the SSADA algorithm (Version: 2015.1.0.90, Optovue, Inc., Fremont, CA, USA), uses blood flow as intrinsic contrast. Indeed, the flow is detected as a variation over time in the speckle pattern formed by interference of light scattered from red blood cells (RBC) and adjacent tissue structure [25,26].

Before imaging, each subject’s pupils were dilated with a combination of 0.5% tropicamide and 10% phenylephrine. Study participants underwent SD-OCT imaging following a protocol that included AngioVue OCT 3D volume set of 3 × 3 mm^2^, consisting of 304 × 304 pixels in the transverse dimension. An internal fixation light was used to center the scanning area.

One FastX (horizontal raster) set and one FastY (vertical raster) set were performed for each acquisition scan. Scans with low quality (i.e., if the subject blinked or if there were significant motion artifacts) were excluded and repeated until good quality was achieved. Three scans for each patient were captured (all with a signal straight index >60) and the scan of best quality was chosen for analysis.

#### 4.3.2. Vascular Layer Segmentation

Vascular retinal layers were visualized and segmented based on the default settings of the automated software algorithm of the XR Avanti AngioVue OCTA [27]. The superficial plexus consists of the capillaries 3 μm below the internal limiting membrane (ILM) to 15 μm below the inner plexiform layer (IPL). The deep plexus extends from 15 μm to 70 μm below the IPL. The choriocapillaris consists of capillaries in a 30 μm thick layer posterior to the retinal pigment epithelium-Bruch membrane junction. The software option to remove projection artifacts from inner vascular plexus in the outer retina was selected. Two observers (LT and RDA) independently checked image quality and excluded poor quality images leading to possible segmentation errors.

#### 4.3.3. Quantitative Vessel Analysis

Objective quantification vessel density was evaluated on the OCTA en face images for each eye using the AngioVue software (Optovue, Inc., Fremont, CA, USA). The flow area was calculated with a user defined circular region of interest (ROI) and a threshold. The area within the ROI with intensities greater than the threshold was calculated. The vessel density was defined as the percentage area occupied by vessels in a circular ROI centered on the center of the foveal avascular zone and with a diameter of 2.5 mm. The AngioVue software automatically splits the ROI into two fields: the foveal area, a central circle with a diameter of 1 mm; and the parafoveal area that constitutes the remaining part inside the ROI [27].

For each patient whole en face (foveal and parafoveal) vessel density, foveal vessel density, parafoveal vessel density, parasuperior and parainferior vessel density in the superficial plexus, deep plexus and in the choriocapillaris were measured.

#### 4.3.4. Qualitative Vessel Analysis

Two independent observers (LT and RDA) subjectively evaluated OCTA in the 3 × 3 mm^2^ scan of best quality. Vascular anomalies were evaluated in terms of vessels caliper (regular or irregular such as telangectasic vascular abnormalities and/or microaneurysms), vessel coarse (regular or irregular such as distorted) and density (normal or rarified).

Perifoveal capillaries were evaluated to disclose disruption or integrity of the perifoveal anastomotic arcades.

Observers were masked about the status of treatment (baseline or post-treatment at different follow up points) of the patient whose images they were analyzing.

#### 4.3.5. Foveal and Parafoveal Retinal Thickness Analysis

Central macular thickness was automatically calculated by the software on the OCTA 3 × 3 mm^2^ volume scan (XR Avanti^®^; Optovue, Inc., Fremont, CA, USA) from ILM to retinal pigment epithelium (RPE). A circular ROI centered on the center of the foveal avascular zone with a diameter of 2.5 mm was considered for retinal thickness analysis: central foveal area (1 mm of diameter) and parafoveal area that constitutes the remaining part inside the ROI (total parafoveal area or temporal, superior, nasal, and inferior quadrants).

#### 4.3.6. Microperimetry

Microperimetry was performed by means of the MP-1 Microperimeter (Nidek Technologies, Padova, Italy), the latter using an infrared fundus camera with a liquid crystal display software-controlled.

All patients were dilated with tropicamide 1% eye drops and, after a pre-test training, 5 min of dark adaptation were performed. The test is routinely carried out with an automated eye tracking system, which provides real-time compensation for eye movements and allows improved presentation of a stimulus at the predefined retinal location. During the test, the patient was encouraged to fix a red ring target, 1° in diameter, on a white monochromatic background at 4 asb. Then, the retinal sensitivity was tested by means of a customized radial grid centered on the fovea and having 77 Goldman III stimuli covering the central 20°. Therefore, the retinal sensitivity can be measured easily because the level of stimulation changes automatically and progressively during the microperimetry test. The stimulus intensity ranged from 0 dB to 20 dB (0 dB corresponded to the strongest signal intensity of 127 cd/m^2^) in 1-dB steps, and the duration of each stimulus was 200 milliseconds. Finally, in order to improve the correlation between microperimetric data with retinal characteristics, results were matched with a color digital retinography obtained with the MP1 color fundus camera. To assess central macular retinal sensitivity, differential light threshold values were compared by calculating the mean of the central 4° of macular area which was averaged automatically by the MP-1 microperimetry software program of mean sensitivity in a polygon.

#### 4.3.7. Treatment

Sustained-release dexamethasone 0.7 mg intravitreal implant (Ozurdex^®^, Allergan, Inc., Irvine, CA, USA) was injected in the vitreous cavity of all patients. All injections were performed in an operation room, and IDI was inserted into the vitreous cavity through the pars plana using a customized, single-use 22-gauge applicator. Patients were treated with a topical ophthalmic antibiotic for 10 days after the treatment.

#### 4.3.8. Main Outcome Measures

Patients were examined at baseline, at 7 days, 30 days, 60 days, 90 days, and 120 days after the IDI implantation.

Main outcome measures were mean VA; microperimetry at 4°; foveal and parafoveal vessel density, and central macular thickness.

#### 4.3.9. Sample Size Determination and Statistical Analysis

The estimation of the number of eyes was based on the main endpoint criteria. A planned sample size of 25 patients was expected to provide 80% power for a two-sided test with significance level of 0.05, assuming an effect size of 17% indifference of BCVA after seven days of implantation with between subjects’ pooled standard deviation of 0.3 logMAR.

The quantitative variables were summarized as mean and standard deviation (SD), qualitative variables as frequency and percentage. A Shapiro–Wilk’s test was performed to evaluate the departures from normality distribution for each variable.

Student’s *t*-test was performed to compare quantitative parameters between controls and DME patients.

Repeated-measures ANOVA with linear trend analysis were performed to evaluate the effect of time on each parameter. Contrast analysis was performed to evaluate differences of each parameters from previous measurement.

The Pearson correlation coefficient (R) was performed to evaluate the correlation among BCVA, microperimetry sensitivity, foveal thickness, and vessel density. The false discovery rate correction (FDR) was used to control the family-wise type I error rate, and an FDR adjusted *p*-value less than 0.05 was determined to be statistically significant. Statistical analysis was performed using IBM^®^ SPSS Statistics v 20.0 software (SPSS Inc., Chicago, IL, USA).

## 5. Conclusions

In conclusion, our study demonstrated that retinal vessel density is reduced in DME eyes with the main involvement of the deep plexus, and that vessel density does not recover after intravitreal steroid treatment. At a morphologic qualitative analysis, deep vessels show a caliber normalization appearing less angiectatic. Partial increase of choriocapillaris density after treatment should be better explored to evaluate the reliability of this finding or attribute it to a limit of OCTA in choriocapillaris analysis in eyes with pathologic overlying retina. Moreover, a longer follow up would be needed to better investigate vascular remodeling in a condition free from macular edema recurrence.

Undoubtedly, OCT angiography represents a fundamental tool in investigating the fine blood vessels in the macula and optic disc, allowing a better comprehension of DME pathogenesis.

## Figures and Tables

**Table 1:** Mean and standard deviation of morphological parameters (central macular thickness and vessel density of superficial capillary plexus, deep capillary plexus and choriocapillaris) and functional parameters (microperimerty and best corrected visual acuity).

Variable	Control	DME	*p-*Value ^a^
CMT (µm)			
Fovea	252.7 ± 20.0	462.1 ± 109.6	<0.001
Parafovea	314.0 ± 13.9	441.3 ± 80.5	<0.001
SCPD (µm)			
Whole	50.2 ± 3.6	40.7 ± 4.5	<0.001
Fovea	32.8 ± 7.8	29.6 ± 5.4	0.263
Parafovea	51.7 ± 4.3	41.3 ± 4.8	<0.001
Parasuperior	52.1 ± 5.2	39.5 ± 6.7	<0.001
Parainferior	51.3 ± 4.0	41.4 ± 6.6	<0.001
DCPD (µm)			
Whole	58.5 ± 3.4	45.1 ± 5.2	<0.001
Fovea	28.5 ± 8.3	18.9 ± 9.2	0.016
Parafovea	61.1 ± 4.3	47.9 ± 5.1	<0.001
Parasuperior	61.1 ± 5.6	45.3 ± 7.5	<0.001
Parainferior	61.1 ± 3.7	47.4 ± 5.8	<0.001
CCD (µm)			
Whole	66.7 ± 1.1	63.5 ± 2.2	<0.001
Fovea	66.0 ± 4.1	60.7 ± 5.8	0.016
Parafovea	65.7 ± 1.7	63.5 ± 2.1	0.010
Parasuperior	65.5 ± 2.2	63.7 ± 2.4	0.042
Parainferior	65.9 ± 1.6	63.3 ± 2.4	0.004
ODVD (µm)			
Whole	54.6 ± 3.6	51.7 ± 4.4	0.164
Inside	42.2 ± 10.6	46.0 ± 10.1	0.431
Peripapillary	61.5 ± 3.7	57.5 ± 4.7	0.021
4° MP (dB)	15.0 ± 1.4	4.9 ± 4.3	<0.001
BCVA (logMAR)	0.1 ± 0.1	0.6 ± 0.1	<0.001

^a^ Student *t*-test DME vs. control. Bolded *p*-value are significant after FDR correction. FDR = false discovery rate; CMT = Central Macular Thickness; SCPD = Superficial capillary plexus density; DCPD = Deep capillary plexus density; CCD = Choriocapillaris density; ODVD = Optic disc vessel density; MP = microperimetry; BCVA = best corrected visual acuity.

**Table 2:** Mean and standard deviation of morphological and functional parameters at baseline and after intravitreal dexamethasone implant.

Variable	Baseline	7 Days	30 Days	60 Days	90 Days	120 Days	*p-*Value ^a^
CMT (µm)							
Fovea	462.1 ± 109.6	347.1 ± 54.7	305.8 ± 53.9 *	286.7 ± 53.9 **	307.8 ± 60.1 *	426.0 ± 95.7	0.001
Parafovea	441.3 ± 80.5	375.3 ± 33.0	349.7 ± 27.3 **	344.9 ± 25.4 *	356.5 ± 31.2	403.7 ± 37.3 *	<0.001
SCPD (µm)							
Whole	40.7 ± 4.5	39.9 ± 4.2 *	41.4 ± 4.9	41.7 ± 4.8	40.6 ± 3.1	41.3 ± 3.6	0.928
Fovea	29.6 ± 5.4	26.7 ± 6.6	26.1 ± 9.4	23.7 ± 9.5 *	27.9 ± 15.1	28.3 ± 3.2	0.311
Parafovea	41.3 ± 4.8	40.2 ± 4.9	41.9 ± 5.5	42.4 ± 5.7	41.0 ± 3.9	41.5 ± 3.9	0.875
Parasuperior	39.5 ± 6.7	39.6 ± 5.6	42.8 ± 6.1	40.0 ± 5.3	41.3 ± 3.5	42.3 ± 3.0	0.491
Parainferior	41.4 ± 6.6	40.9 ± 5.1	43.1 ± 4.8	40.9 ± 4.5 *	41.5 ± 5.4	41.8 ± 4.4	0.296
DCPD (µm)							
Whole	45.1 ± 5.2	46.4 ± 5.0	48.3 ± 4.3	46.9 ± 4.3	42.8 ± 6.9	47.2 ± 6.3	0.210
Fovea	18.9 ± 9.2	23.3 ± 12.0	26.7 ± 10.0	24.5 ± 6.8	26.7 ± 6.9	20.9 ± 8.7	0.205
Parafovea	47.9 ± 5.1	48.2 ± 5.3	50.6 ± 5.1	48.7 ± 5.2	44.5 ± 6.4	49.1 ± 7.4	0.144
Parasuperior	45.3 ± 7.5	48.4 ± 5.4	50.9 ± 4.9	49.6 ± 5.2 *	45.4 ± 5.9	50.8 ± 4.9	0.110
Parainferior	47.4 ± 5.8	48.1 ± 5.7	50.2 ± 5.4	47.7 ± 5.5	43.7 ± 7.0	47.5 ± 10.1	0.269
CCD (µm)							
Whole	63.5 ± 2.2	63.9 ± 1.5	64.8 ± 1.8	65.4 ± 1.6	64.6 ± 1.7	61.6 ± 6.3	0.414
Fovea	60.7 ± 5.8	61.4 ± 6.1	65.8 ± 2.9	64.7 ± 5.8	63.9 ± 5.6	60.9 ± 8.5	0.188
Parafovea	63.5 ± 2.1	64.0 ± 1.4	64.5 ± 2.0	65.3 ± 1.5	64.1 ± 1.4	60.8 ± 6.8	0.353
Parasuperior	63.7 ± 2.4	64.2 ± 1.5	64.5 ± 2.1 *	65.9 ± 1.7	63.9 ± 1.5	60.6 ± 7.3	0.261
Parainferior	63.3 ± 2.4	63.8 ± 1.9	64.4 ± 2.2	64.6 ± 1.7	64.3 ± 1.8	61.0 ± 6.4	0.497
ODVD (µm)							
Whole	51.7 ± 4.4	51.0 ± 3.1	50.2 ± 4.5	50.2 ± 2.9	50.4 ± 3.6	49.2 ± 3.8	0.573
Inside	46.0 ± 10.1	45.8 ± 10.5	42.9 ± 9.7	45.0 ± 8.8	42.8 ± 10.2	42.7 ± 10.4	0.452
Peripapillary	57.5 ± 4.3	56.5 ± 4.4	55.2 ± 5.8	55.5 ± 3.7	56.4 ± 4.7	54.5 ± 5.8	0.363
4° MP (dB)	4,9 ± 4,3	6.5 ± 4.6 **	7.5 ± 4.7 **	7.4 ± 5.1	7.1 ± 5.4	5.5 ± 5.6	0.611
BCVA (logMAR)	0.6 ± 0	0.5 ± 0.5	0.4 ± 0.2	0.3 ± 0.2	0.3 ± 0.2	0.3 ± 0.2	0.001

^a^ Repeated-measures ANOVA with linear trend analysis. Bolded *p*-value are significant after FDR correction. * *p* < 0.05; ** *p* < 0.01 contrast analysis vs. previous measure. FDR = false discovery rate; CMT = Central Macular Thickness; SCPD = Superficial capillary plexus density; DCPD = Deep capillary plexus density; CCD = Choriocapillaris density; ODVD = Optic disc vessel density; MP = microperimetry; BCVA = best corrected visual acuity.

**Table 3:** Pearson’s Correlation coefficient among thickness, density, and functional parameters expressed as relative variation from baseline to 120 days.

Variable	CMT Fovea	CMT Parafovea
SCPD		
Whole	−0.490	−0.121
Fovea	−0.322	0.224
Parafovea	−0.470	0.034
DCPD		
Whole	0.187	0.616
Fovea	−0.220	0.132
Parafovea	0.308	0.689
CCD		
Whole	−0.146	0.048
Fovea	−0.724 **	−0.799 **
Parafovea	−0.222	0.078
ODVD		
Whole	−0.243	−0.131
Inside	−0.044	0.220
Peripapillary	−0.543	−0.257
4° MP	−0.028	0.251
BCVA	0.123	−0.365

* *p* < 0.05; ** *p* < 0.01 after FDR correction. FDR = false discovery rate, CMT = Central Macular Thickness; SCPD = Superficial capillary plexus density; DCPD = Deep capillary plexus density; CCD = Choriocapillaris density; ODVD = Optic disc vessel density; MP = microperimetry; BCVA = best corrected visual acuity.
